# Cross-Sectional Study of Factors Influencing Perceived Threat and Stress among the Arab Minority in Israel during the COVID-19 Pandemic

**DOI:** 10.3390/ijerph191610326

**Published:** 2022-08-19

**Authors:** Ola Ali-Saleh, Ofra Halperin

**Affiliations:** Department of Nursing, Max Stern Academic College of Emek Yezreel, Emek Yezreel 1930600, Israel

**Keywords:** COVID-19, locus of control, coping strategies, loneliness, perceived threat, stress

## Abstract

This study aimed to examine the impact of the locus of control, coping strategies and loneliness on perceived threat and stress among the Arab minority in Israel during the first COVID-19 closure. This was a cross-sectional online study, with 486 participants who completed a questionnaire measuring the study variables during the period of 28–31 March 2020. Statistical analyses included *t*-tests and analyses of variance. Means, standard deviations and intercorrelations for the study variables were calculated. The results revealed a moderate-low level of stress and a moderate-high level of perceived threat. Higher stress was related to higher perceived threat, a greater external locus of control, lower problem-focused coping, higher emotion-focused coping and social support-seeking and higher loneliness. Perceived threat was positively related to both problem-focused coping and emotion-focused coping. The results show that the Arab population in Israel coped as a strong minority group. This study contributes to our understanding of how minority groups cope in the current epidemic and to the identification of effective strategies for reducing stress during this challenging period. The study’s results may help devise intervention programs that foster more effective coping capabilities among this and other minority populations.

## 1. Introduction

The World Health Organization has declared the COVID-19 outbreak a global state of emergency, raising concerns regarding public health [1]. This pandemic threatens and challenges mental and physical public health, as well as the health systems in many countries [2].

Outbreaks of epidemics are known to be extremely stressful events. They are unpredictable, vague, and completely uncertain [3], and are often associated with psychological distress and symptoms of mental illness [4]. Evidence suggests that during outbreaks of infectious diseases, people may experience symptoms of psychosis, trauma, suicidal thoughts and panic [5], depression and sometimes post-traumatic stress disorder, as well as anxiety [6]. Moreover, almost one in five people suffers from depressive symptoms, sleep problems and stress [6]. Symptoms such as anxiety, depression, fear, stress, and sleep problems have been reported more frequently during the COVID-19 epidemic [7], as have negative behaviors such as increased alcohol and tobacco consumption [8].

The COVID-19 epidemic is associated with feelings of helplessness as well as loss of a basic sense of safety, security, financial stability and the ability to imagine a brighter future. Disease threat is also associated with fear of infection, inability to predict the disease, severe disease complications [4], high mortality rates, over-diagnosis of infections and concerns about the future [9]. Fear of the unknown, uncertainty [6,8] and the inability to control the disease and risk severity may increase feelings of stress and anxiety [4].

In these and other difficult and stressful situations, a sense of control may promote better coping and mental well-being [10]. Studies have found a negative association between the locus of control and the development of PTSD among adolescents who have experienced a natural disaster, with the internal locus of control serving as a strong protective factor against PTSD symptoms [11]. When people deal with stressful life events, their perceived control over outcomes has been shown to be positively related to a sense of well-being and quality of life, and negatively related to emotional distress [12].

To slow the spread of COVID-19 and reduce the burden on the health system, the Center for Disease Control and Prevention (CDC) has recommended physical (social) distancing, self-closure and the isolation of infected individuals [13]. As in other countries in the world, Israel has issued many instructions and guidelines to the population. On 18 March 2020, the Israeli government imposed a closure and instructed citizens to distance themselves from physical and social interactions [14].

Social distancing is one of the most successful strategies used in the past to slow or prevent community infection during epidemics. Nevertheless, losing the ability to communicate physically, face-to-face, with people, and to maintain social ties, may raise stress levels significantly [15] and increase feelings of loneliness [9,16]. A recent study found that loneliness was associated with an 18% increased risk of all-cause mortality among adults living alone [17]. Loneliness is also a risk factor for many mental disorders, including depression, anxiety, adjustment disorder, chronic stress, insomnia and even dementia; and for chronic diseases, including hypertension [18], cardiovascular disease and stroke [19]. Moreover, loneliness is associated with an increased risk of morbidity and mortality [20].

Coping is defined as the actual effort made in attempting to render a perceived stressor tolerable and minimize the distress induced by the situation [21]. Coping entails the thoughts and behaviors used to manage the demands of a specific person-environment transaction relevant to the individual’s well-being. Coping has two major functions: dealing with the problem causing the distress (problem-focused coping) and regulating emotion (emotion-focused coping) [21].

People’s responses to stress during the pandemic can depend on their background, social support from family or friends, financial situation, health and emotional background, and the community in which they live. Taha et al. [22] found that a greater intolerance of uncertainty predicted low levels of problem-focused coping and more reports of anxiety. Additionally, individuals with a high intolerance for uncertainty were more likely to perceive the pandemic as threatening and more apt to use emotion-focused coping strategies, with both predicting elevated levels of anxiety. Both problem-focused and emotion-focused coping strategies have been identified in the current epidemic. People who used problem-focused coping invented new leisure and social activities. While social distancing kept people apart, social media became the medium of choice for reuniting with relatives and long-lost friends for those seeking a semblance of human touch. Emotion-focused coping strategies designed to regulate people’s emotions and distance them from the source of stress were also prevalent. Leisure offered an escape from the stresses of COVID-19, unemployment and the boredom of home confinement [23].

Research shows that social variables, such as race and ethnicity, education and socioeconomic status, can explain variations in how different groups are affected by disasters [24]. Disaster-related stress is higher among people with lower levels of education and income, minority groups and societies with more minors, reflecting the vulnerability of each of these groups to specific mental and social pressures [25].

In Israel, Arab citizens are an ethnic minority, constituting 21% of the population. Ninety percent live in homogeneous peripheral localities ranked in lower socioeconomic clusters [26], which tend to be overcrowded [27]. Studies point to socioeconomic and health inequality among Israel’s Arab population. Before COVID-19, Arabs were over-represented in all measures of poverty, distress, unemployment and school dropout rates [28]. Gaps in family size, education, employment and wages caused large SES discrepancies between Arabs and Jews. In 2016, 53% of Arab families lived in poverty, compared with 14% of Jewish families, while almost 66% of Arab children lived in poverty, compared with 20% of Jewish children [29]. Arab men who smoke have a higher incidence of chronic lower respiratory diseases; Arabs of both genders have a higher incidence of diabetes, hypertension and cardiovascular disease than the Jewish population [27].

The subjective health status serves as a measure of health in many studies of health-related issues, as it is a good predictor of mortality, morbidity, and use of health services. Arabs tend to evaluate their health as better than Jews, although their life expectancy is lower and their morbidity and mortality are higher [30].

The Arab population in Israel faces major challenges in meeting the Health Ministry’s COVID-19 guidelines. Hence, it is likely to experience more stress due to the onset of COVID-19. Kimhi et al. [31] found a higher level of distress and a lower level of resilience and well-being among Israeli Arabs. Since, to the best of our knowledge, there are not sufficient studies on the understanding of perceived threat and stress among the Arab minority in Israel, this establishes the need for more studies.

The current study aimed to investigate the locus of control, coping strategies and loneliness as factors influencing threat perceptions and stress among the Arab minority in Israel at the beginning of the first COVID-19 closure. It seeks to examine how this unique population is coping with COVID-19 and identify the characteristics contributing to the degree of stress they experience. The study will make it possible to learn how minority groups cope in the current epidemic, with the goal of identifying strategies that may be particularly effective in reducing stress during this challenging period.

## 2. Materials and Methods

The current study is a cross-sectional online study conducted among the Arab population in Israel during the first COVID-19 closure. The questionnaire was distributed via social networks, mainly Facebook and WhatsApp, during the period of 28–31 March 2020. The questionnaire consisted of 90 questions and took approximately twenty minutes to complete. Participation was voluntary and no permutations were offered (snowball sampling). Inclusion criteria were Arabs over 18 years of age and exclusion criteria were non- Arabs or those under the age of 18. The Institutional Review Board and Ethics Committee of The Max Stern Yezreel Valley College approved the study.

### 2.1. Instruments

All questionnaires were translated into Arabic by English-language professionals and then back-translated into English. All questionnaires are validated on the Arab population.

Perceived Stress Scale (PSS)—Cohen et al., (1994) [32]: Participants answer on a 4-point scale (1 = never; 4 = often), with a high score reflecting a high stress level. The questionnaire score is calculated by averaging the items. Cronbach’s alpha was α = 0.89. We added four items [15,16,17,18] related to COVID-19, all with good internal consistency: (α = 0.79). The questionnaire includes eight positively worded items (4, 5, 6, 7, 8, 9, 10 and 13) and ten negatively worded items (items 1, 2, 3, 11, 12, 14, 15, 16, 17 and 18). Higher scores indicate higher levels of perceived pressure.

The Locus of Control Scale is a valid and reliable Hebrew version of Rotter’s (1966) I-E locus of control scale, which is the final version used in most locus of control studies. The questionnaire includes ten sentences referring to respondents’ level of control over their lives, divided into an ordinal scale of five categories (5 = to a very large extent, 3 = to a small extent and 1 = not at all). For example: “From my experience, I know that what is supposed to happen will indeed happen”. Scores range from 10 to 50, with higher scores indicating that the focus of control tends to be internal. The internal reliability index is reversed for items 1, 4, 6, 7 and 10.

The coping questionnaire was developed by Carver et al., (1989) [33]. Here, we relied on the abbreviated Hebrew version proposed by Ben-Zur and Zeidner (1993). This version includes two items for each coping strategy (planning, seeking emotional support, positive vision and growth, seeking instrumental support, active coping, venting emotions, accepting the situation, suppressing competing actions, restraint, humor, mental detachment, behavioral detachment, religion, denial and alcohol/drug use). A factor analysis yielded two scales: (1) problem-focused coping (α = 0.71), including items 7, 10, 14 and 2 (Table 2) and items 3 and 5 (Table 3); (2) emotion-focused coping (α = 0.77), including the remaining items. Total scores were calculated as the item means, so that higher scores represent higher use of each style, with results ranging from 0–3.

The questionnaire examining feelings of loneliness (Friedman, 1985) included 20 items examining social relationship satisfaction. Ten items were positively worded to indicate a lack of loneliness, e.g., “I do not feel lonely”. Ten items were negatively worded to indicate loneliness, e.g., “I have no one to turn to”. Participants responded to each statement on a scale from 1 (not at all) to 4 (to a large extent). The questionnaire score was calculated according to the sum of scores after reversal of positively worded items. The sum of the scores ranged from 20 (low loneliness) to 80 (high loneliness). Item reliability was tested using Cronbach’s alpha coefficient and found to be very high (α = 0.85).

### 2.2. Data Analysis

Data were analyzed using SPSS ver. 26 (IBM Corp., Armonk, NY, USA) Background variables were described using frequencies and percentages, means and standard deviations. Relationships between the dependent variables and the background variables were analyzed using *t*-tests and analyses of variance. Means, standard deviations and Pearson correlations for the study variables were calculated.

The study model was examined with a path analysis, using AMOS ver. 26 (IBM SPSS, Chicago, IL, USA). Background variables served as control variables and were binary. All continuous variables were standardized. The control variables and the independent variables were allowed to correlate within themselves. χ^2^, NFI, NNFI, CFI and RMSEA were used as model fit measures. The model was calculated using bootstrapping with 200 samples and bias corrected confidence interval, with 95% confidence level.

The population in Israel consists of 1.995 million Arabs, which constitute 21.1% of the total population. As the questionnaire was distributed through social networks, the distribution was limited and a calculation was made for a proper sample size. Sample size was calculated with G*Power 3.1 [34]. Considering a multiple regression analysis with low to moderate effect size of f^2^ = 0.10, α = 0.05, ten predictors (four control variables, five independent variables and one mediator) and power of 0.95, a sample size of 254 respondents was required.

## 3. Results

### Sample Characteristics

Participants in this study were 486 Israeli Arabs, 102 men (21%) and 384 women (79%), all adults of working age, as shown in Table 1. Most were Muslim, living in rural communities and were mainly religious or partly religious. Most were married or in a steady relationship and had children. Further, most had an academic education and usually reported their health as good. Most participants were employed, usually outside the home, and about 30% of them were forced to stop working due to COVID-19.

Stress was moderate-low (*M* = 2.18, *SD* = 0.47, range 1–4), whereas the perceived COVID-19 threat was moderate-high (*M* = 3.15, *SD* = 0.62, range 1–4). Stress was higher for women than for men (*M* = 2.23, *SD* = 0.47 vs. *M* = 2.02, *SD* = 0.43) (*t*(483) = 3.98, *p* < 0.001), as was the perceived threat (*M* = 3.20, *SD* = 0.59 vs. *M* = 2.98, *SD* = 0.70) (*t*(141.61) = 2.87, *p* = 0.005). Stress was higher among younger participants (age 18–25: *M* = 2.32, *SD* = 0.45, and age 26–45: *M* = 2.22, *SD* = 0.47) than older ones (age 46–66: *M* = 2.06, *SD* = 0.46) (*F*(2, 482) = 8.45, *p* < 0.001, η^2^ = 0.034). Further, stress was higher for participants with a high school or professional education (*M* = 2.28, *SD* = 0.51) than for participants with an academic education (*M* = 2.14, *SD* = 0.44) (*t*(483) = 3.27, *p* = 0.001). Moreover, stress was higher for unemployed (*M* = 2.33, *SD* = 0.53) than for employed participants (*M* = 2.16, *SD* = 0.45) (*t*(483) = 2.89, *p* = 0.004).

The means in Table 2 reveal a moderate-low stress level and a moderate-high perceived threat level. The internal locus of control was moderate-high, as was problem-focused coping. Emotion-focused coping was rather low, support-seeking was moderate-low and the sense of loneliness was moderate-low. Stress was positively associated with the perceived threat, emotion-focused coping, support-seeking and sense of loneliness and negatively associated with the internal locus of control and problem-focused coping. Perceived threat was positively related to all coping styles and negatively related to the internal locus of control. The internal locus of control usually exhibited a negative relation to emotion-focused coping and a sense of loneliness. Both problem-focused and emotion-focused coping were positively associated with support-seeking. Nevertheless, while problem-focused coping showed a negative relationship with loneliness, the opposite relationship was found for emotion-focused coping.

The study model was examined with a path analysis, using AMOS ver. 26 (IBM SPSS, Chicago, IL, USA). Control variables were gender (1—male, 0—female), age (1: 46 to 66 years, 0: 18 to 45 years), education level (1—academic, 0—lower than academic) and employment status (1—employed, 0—unemployed). Independent variables were the locus of control, coping strategies and a sense of loneliness, the mediating variable was perceived threat and the dependent variable was experienced stress.

**Figure 1:**
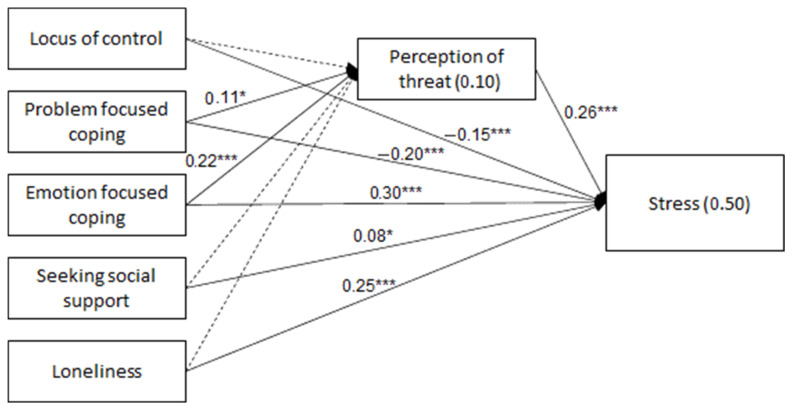
Path Analysis for Locus of Control, Coping Strategies, Sense of Loneliness, Perceived Threat and Experienced Stress. * *p* < 0.05, *** *p* < 0.001.

The model was found to fit the data: χ^2^(20) = 22.45, *p* = 0.316, NFI = 0.973, NNFI = 0.991, CFI = 0.997, RMSEA = 0.016. As shown in Table 3 and Figure 1, experienced stress was positively related to perceived threat, negatively related to the internal locus of control and problem-focused coping and positively related to emotion-focused coping, seeking social support and a sense of loneliness. That is, higher stress was associated with higher perceived threat, a greater external locus of control, lower problem-focused coping, higher emotion-focused coping and social support-seeking and a higher sense of loneliness. Perceived threat was positively associated with both problem-focused and emotion-focused coping, meaning that higher problem-focused and emotion-focused coping were related to a greater perceived threat.

These results reveal that perceived threat may mediate how problem-focused and emotion-focused coping are related to experienced stress. The two indirect relationships were significant: problem-focused coping (standardized indirect effect = 0.029, *SE* = 0.016, *p* = 0.024, 95% CI = 0.004, 0.082) and emotion-focused coping (standardized indirect effect = 0.059, *SE* = 0.015, *p* = 0.012, 95% CI = 0.031, 0.090). That is, higher problem-focused coping was associated with higher perceived threat, which was then related to higher experienced stress. Yet higher problem-focused coping was directly related to lower experienced stress. Higher emotion-focused coping was related to higher perceived threat, which was then related to higher experienced stress. The direct relationship between emotion-focused coping and experienced stress was also positive.

To summarize, the direct relationship between the internal locus of control and stress was negative. The direct relationship between problem-focused coping and stress was negative, while the indirect relationship mediated by perceived threat was positive. Both the direct and indirect relationships between emotion-focused coping and stress were positive. Finally, the direct relationships between seeking social support and stress and between a sense of loneliness and stress were positive.

## 4. Discussion

The current study sought to examine coping strategies, the locus of control and feelings of loneliness as factors influencing the degree of stress and perceived COVID-19 threat among the Arab population as an ethnic minority in Israel at the beginning of the first COVID-19 closure.

The perceived COVID-19 threat was moderate-high. The Arab population in Israel faces many challenges dealing with the COVID-19 epidemic threat in general and with implementing physical and social distancing guidelines in particular, which may increase the sense of threat from the disease and its consequences. In addition, the Arab population suffers from overcrowding and a shortage of housing and most Arab localities are classified as belonging to lower socioeconomic clusters. The average number of persons in an Arab family is 4.6 and about 28% of Arab families number six or more persons. Moreover, these large families include adults, young people and a large proportion of children who come into close contact with many peers every day. The Arab society is marked by multiple family and social events which people find difficult to give up. Finally, the collectivist nature of Arab culture is dominant even at times of crisis. Therefore, the guidelines may be perceived as contradictory to their lifestyle, leading to a tendency to violate them. Their living conditions may increase their risk perceptions and sense of threat that the disease may actually endanger many lives, especially the elderly, chronically ill or risk groups. In addition, we found that feeling threatened by the disease increases feelings of stress. It is also possible that the living conditions in Arab society and the overcrowding in their social relationships lead to reports of a low level of loneliness.

A study in Italy during the current epidemic found that a high level of stress was caused, among other things, by fear of infection and worries that a relative would get sick [35]. In outbreaks of widespread epidemics, implementing social isolation and quarantine in an attempt at containment may also lead to a high level of mental distress [36]. Feelings of uncertainty have direct implications on the population’s daily life and mental health [37]. 

In this study, the internal locus of control was moderate-high, as was problem-focused coping. Higher problem-focused coping was related to higher perceived threat, which was then related to higher experienced stress. Yet higher problem-focused coping was directly related to lower experienced stress. Studies found that increased use of emotion-focused coping correlates positively with psychological distress [38]. In contrast, a high level of problem-focused strategies exhibits weak correlations with distress, but is related to positive affect [39]. In a study with a Norwegian- and German-speaking population, the major finding was that both aspects of the LoC (internal and external) showed substantial moderation effects on the relationship between COVID-19 stress and general mental distress during the early months of the COVID-19 pandemic [40]. Ethnic minority groups, such as African-, Asian- and Mexican-Americans, tended to hold a more external locus of control [41] than European-Americans. An orientation towards the external locus of control seems consistent with fatalistic beliefs that prevail in many Asian cultures [42]. In African-American cultures, having a more external locus of control orientation may be a way of dealing with the disadvantages of poverty, unemployment and racial discrimination [43].

For many, the uncertainty surrounding COVID-19 is the hardest to handle. People are not sure how they will be affected or how bad things might get, making it all too easy to catastrophize and spiral into a sense of overwhelming dread and panic. People with an external locus of control believe that events are not under their control. These people do not fight to protect themselves from psychological harm, believing they cannot change the situation because someone else is in charge [44].

The Arab population in Israel faces barriers to accessing information and difficulties in adopting social distancing measures. A significant delay in disseminating instructions in Arabic led to severe gaps in the level and scope of the information reaching much of the Arab public. The large numbers of Arab doctors, nurses, pharmacists and other health and welfare providers may help raise public awareness in the Arab sector regarding the importance of following governmental instructions.

Stress was moderate-low and was higher among young unemployed women with a high school education only. This is in line with the literature, where individual differences are explained by the degree of stress individuals endure, with women usually more affected by stressors than men [45]. Women and men differ in their exposure and reactions to stressors, in that women experience more chronic stressors than men and consider stressors to be more threatening [45]. Previous studies have demonstrated that the perceived mental stress may intensify feelings of loneliness. For instance, Yarcheski et al. [46] reported a significant positive correlation between perceived mental stress and loneliness. Similarly, Brown et al. [47] have also demonstrated positive associations between perceived mental stress and feelings of loneliness.

Stress was positively connected with perceived threat and emotion-focused coping, which affect feelings associated with the stressor, seeking social support and loneliness. Yet, stress was negatively connected with the internal locus of control and problem-focused coping, which affect the stressor itself. This is in line with other studies that found that social support can only be of help when it conforms to the coping strategies that are most adequate in the stressful situation [48]. People who more frequently use specific emotion-focused coping strategies (e.g., focusing on and venting emotions, denial, behavioral and mental disengagement and seeking emotional social support) experience more stress [49].

As in other studies, in this study, perceived threat was positively associated with all the coping mechanisms [50]. The personal choice of coping strategies is determined by personality traits and type, social context and the nature of the stressor involved [49]. Nevertheless, perceived threat was negatively associated with the internal locus of control. These results conform to other studies that found significant relationships between perceived threat and depression only among participants who reported low levels of an internal locus of control [51]. Clinical implications emphasize the importance of cognitive interventions aimed at challenging perceived threat and control as a mean of reducing depression [52]. Our findings show that perceived threat may mediate how problem-focused and emotion-focused coping are related to stress. Such information may prove essential to public health and health promotion in the face of communicable diseases.

The study’s limitations include its cross-sectional design and its homogeneous sample. Future studies using a longitudinal design and heterogeneous samples should be carried out to further validate the current results.

## 5. Conclusions

The present study highlights the importance of specific coping strategies in managing the stress of perceived health threats. Our results expand understanding regarding the use of coping strategies in relation to the internal locus of control. COVID-19 has reminded us that our health can be threatened. Understanding the psychological and behavioral reactions to health threats may inform health messages and inspire more effective and productive campaigns to reduce the damaging consequences of such diseases on our social well-being. The study’s results show how the Arab population in Israel copes as a strong minority group and may help devise intervention programs fostering more effective coping capabilities among this and other minority populations. Moreover, during crises, policymakers need to increase support for minority populations, for example, by promoting mental health and building sensitive and tailored intervention strategies to help reduce stress. Identifying the mental challenges of minority groups during disasters can lead to understanding how to reduce these negative effects and improve coping abilities during disasters.

## Figures and Tables

**Table 1:** Background Characteristics (*n* = 486).

	Variable	
Gender	Male (%)	102 (21.0)
	Female (%)	384 (79.0)
Age	18–25 (%)	56 (11.5)
	26–45 (%)	281 (57.8)
	46–65 (%)	149 (30.7)
Ethnicity	Muslim (%)	426 (87.7)
	Christian (%)	47 (9.7)
	Other (%)	13 (2.6)
Place of living	Rural (%)	363 (74.7)
	Urban (%)	123 (25.3)
Religiosity	Secular (%)	61 (12.6)
	Partly religious (%)	220 (45.5)
	Religious (%)	203 (41.9)
Marital status	Married/in a steady relationship (%)	397 (81.7)
	Single (%)	71 (14.6)
	Divorced/Widowed (%)	18 (3.7)
Children	Yes (%)	383 (78.8)
Number of children	Mean number of children (*SD*), range	3.07 (1.06), 1–7
Education	Elementary (%)	4 (0.8)
	High school (%)	77 (15.8)
	Professional (%)	76 (15.6)
	Academic (%)	329 (67.7)
Health	Good (%)	437 (89.9)
	Not good (%)	49 (10.1)
Employment	Employed (%)	412 (84.8)
	Unemployed (%)	74 (15.2)
Usual place of work (*n* = 412)	Outside the home (%)	322 (78.2)
	At home (%)	90 (21.8)
Leave of absence during COVID (*n* = 412)	Yes (%)	124 (30.1)
	No (%)	288 (69.9)

**Table 2:** Means, Standard Deviations and Intercorrelations Between Study Variables (*n* = 486).

	M (SD)	2.	3.	4.	5.	6.	7.
1. Stress (1–4)	2.18 (0.47)	0.37 ***	−0.38 ***	−0.22 ***	0.54 ***	0.14 **	0.47 ***
2. Perceived threat (1–4)	3.15 (0.62)		−0.14 **	0.12 **	0.25 ***	0.15 ***	0.06
3. Locus of control (1–5)	3.68 (0.64)			0.14 **	−0.32 ***	−0.09 *	−0.24 ***
4. Problem-focused coping (0–3)	1.96 (0.44)				−0.01	0.30 ***	−0.21 ***
5. Emotion-focused coping (0–3)	0.87 (0.38)					0.30 ***	0.40 ***
6. Support-seeking (0–3)	1.16 (0.56)						−0.12 **
7. Loneliness (1–4)	2.01 (0.44)						

* *p* < 0.05, ** *p* < 0.01, *** *p* < 0.001.

**Table 3:** Direct Relationships between Locus of Control, Coping Strategies, Sense of Loneliness, Perceived Threat and Experienced Stress (*n* = 486).

DV (R^2^)	IV	β	SE	*p*
Perceived threat (0.10)	Locus of control	−0.08	0.05	0.079
	Problem-focused coping	0.11	0.05	0.018
	Emotion-focused coping	0.22	0.05	<0.001
	Seeking social support	0.03	0.05	0.595
	Loneliness	−0.04	0.05	0.459
Experienced stress (0.50)	Locus of control	−0.15	0.03	<0.001
	Problem-focused coping	−0.20	0.03	<0.001
	Emotion-focused coping	0.30	0.04	<0.001
	Seeking social support	0.08	0.04	0.040
	Loneliness	0.25	0.04	<0.001
	Perceived threat	0.26	0.03	<0.001

Note. DV—dependent variable, IV—independent variable.

## Data Availability

The datasets used and/or analyzed during the current study are available from the corresponding author upon reasonable request.
